# Neuropsychological Predictors of Treatment Dropout Among Intimate Partner Violence Perpetrators

**DOI:** 10.3390/jintelligence14080164

**Published:** 2026-08-01

**Authors:** Ángel Romero-Martínez, Carolina Sarrate-Costa, Marisol Lila, Luis Moya-Albiol

**Affiliations:** 1 Department of Psychobiology, Faculty of Psychology, University of Valencia, 46010 Valencia, Spain; carolina.sarrate@uv.es (C.S.-C.); luis.moya@uv.es (L.M.-A.); 2 Department of Social Psychology, Faculty of Psychology, University of Valencia, 46010 Valencia, Spain; marisol.lila@uv.es

**Keywords:** dropout, executive functioning, intelligence quotient, intervention, intimate partner violence

## Abstract

**Background**: The interplay between intervention dropout and increased recidivism among men convicted of intimate partner violence (IPV) underscores the need for a model that investigates the underlying variables associated with IPV perpetrator dropout. While sociodemographic factors are traditional predictors of dropout, the potential role of cognitive functioning remains insufficiently researched. This study examined whether certain neuropsychological domains, previously signaled by the scientific literature, including general intelligence, cognitive flexibility, planning abilities, verbal fluency, attention, and working memory, were associated with treatment attrition among IPV perpetrators. **Methods**: Participants (*n* = 309) underwent a comprehensive neuropsychological battery (K-BIT, WCST, BADS, CPT-III, and WAIS-III) prior to a court-mandated program. **Results**: The final regression model indicated that higher perseverative errors (WCST), lower nonverbal IQ, impaired planning abilities (Key Search Test), and a higher number of omissions in the CPT-III were significantly associated with dropout while controlling for alcohol misuse and sociodemographic variables. The overall predictive power of the neuropsychological model was moderate, offering a valuable, although complementary, framework to traditional models that only focus on sociodemographic variables. **Conclusions**: Early screening of neuropsychological variables could potentially help predict which individuals are at high risk of dropout. While these observational findings require external validation, integrating cognitive considerations into intervention planning warrants further investigation to understand their association with treatment adherence.

## 1. Introduction

Intimate partner violence (IPV) is a multifaceted social and public health phenomenon, the mitigation of which depends on, among other factors, the efficacy of court-mandated batterer intervention programs (World Health Organization, 2025). In fact, IPV perpetrators who successfully complete court-mandated interventions exhibit a significantly lower risk of recidivism compared to those who drop out. Unfortunately, overall dropout rates in current interventions are significant, ranging from 9 to 67% (Cunha et al., 2024). Therefore, dropout could be considered a primary obstacle to rehabilitation and a significant predictor of subsequent recidivism (Jewell & Wormith, 2010; D. B. Wilson et al., 2021). The reduced reoffending observed in IPV perpetrators who completed the intervention could be attributed, at least in part, to the internalization of non-violent conflict resolution strategies and the cognitive restructuring of patriarchal beliefs facilitated throughout the program. Hence, treatment completion serves as a critical threshold where the acquisition of self-regulation skills and emotional management effectively mitigates the propensity for future violence (Arce et al., 2020; Oğuztüzün et al., 2023; D. B. Wilson et al., 2021). Furthermore, an individual’s neuropsychological capacity to process and apply these therapeutic insights is central, making the prevention of premature dropout an essential prerequisite for reducing the long-term risk of recidivism (McDonagh et al., 2025).

Traditionally, research has sought to explain dropout by focusing on several sociodemographic variables. In this regard, three meta-analyses identified younger age, lower educational level, employment status, and unstable housing, along with alcohol issues, as significant predictors of a participant’s failure to complete court-mandated interventions (Cunha et al., 2024; Jewell & Wormith, 2010; D. B. Wilson et al., 2021). These authors hypothesized that the presence of co-occurring life stressors, such as financial instability and lack of social support, may diminish the perpetrator’s perceived investment in the therapeutic process, leading to early disengagement. In addition, treatment programs show significantly lower success rates (and higher risk of recidivism) for IPV perpetrators with high baselines of psychological or sexual violence compared to those with a high presence of physical violence before starting intervention programs (Oğuztüzün et al., 2023). While these sociodemographic markers and type of violence provide a valuable framework for identifying high-risk populations, they often fail to account for the underlying cognitive mechanisms that govern behavioral persistence and self-regulation. Consequently, there is an increasing shift toward integrating these external factors with internal neuropsychological profiles, such as intellectual capacity and executive functioning, to develop a more comprehensive understanding of why certain individuals remain resistant to standardized intervention models (Pinto et al., 2010; Romero-Martínez et al., 2024).

Among the neuropsychological variables that appear particularly relevant to dropout among IPV perpetrators, low intelligence quotient (IQ), cognitive flexibility, and working memory were significantly associated with treatment dropout (Pinto et al., 2010; Romero-Martínez et al., 2023). Intelligence, defined as a general mental ability that involves the capacity to reason, solve problems, and learn from experience measured in terms of IQ (Gottfredson, 1997), and cognitive flexibility, defined as the ability to adapt cognitive strategies to new and unexpected environmental stimuli (Diamond, 2013), together with working memory alterations, provide the necessary framework for an individual to internalize complex socioemotional information and inhibit maladaptive responses (Goldstein & Naglieri, 2014). These research studies suggest that IPV perpetrators who present low IQ, a higher number of perseverative errors and an inability to adapt their responses to changes in task rules, and key indicators of low cognitive flexibility, are significantly more likely to drop out of intervention programs prematurely. This mental rigidity (or low cognitive flexibility) together with low IQ and working memory functioning not only hinders the learning of new conflict-resolution strategies during therapy, but also stands as a critical predictor of treatment dropout before therapeutic goals are met. Moreover, these cognitive alterations were linked to alcohol misuse, given that heavy drinkers (or IPV perpetrators who presented a higher likelihood of being diagnosed with alcohol use disorder) often show the worst performance on these cognitive domains. This combination creates a high-risk profile: alcohol consumption exacerbates cognitive impairments, leading to a rigid mental state that prevents offenders from adapting to the program’s demands or internalizing new conflict-resolution skills, which may, in part, explain their premature dropout (Romero-Martínez et al., 2023, 2024). However, these studies did not explore other relevant cognitive domains that could further explain dropout among IPV perpetrators.

The neuropsychological alterations observed in IPV perpetrators that explain treatment dropout partly align with findings from other studies examining determinants of treatment dropout in high-risk populations other than IPV perpetrators. In fact, in individuals with drug use disorders, the low IQ explained treatment dropout (Adams, 2009). Other studies also found that planning abilities, attention, and verbal fluency (phonological and semantic) were directly associated with discontinuation of treatment or dropout in individuals with HIV, patients with bipolar disorder, drug use disorder, or schizophrenia (Becker et al., 2011; Fagan et al., 2015; Stimmel et al., 2018; Wojtalik et al., 2023; Zhu et al., 2023). In this sense, a diminished planning capacity, understood as the ability to identify and organize the sequence of steps required to achieve a specific goal (Lezak et al., 2012), and impaired attention and working memory, which are the systems responsible for the temporary storage and manipulation of information necessary for complex cognitive tasks (Baddeley, 2000), affect an individual’s ability to organize goal-directed actions and foresee the negative consequences of both violent behavior and the decision to drop out of treatment. Furthermore, deficits in verbal phonological fluency (the retrieval of words based on orthographic constraints) and semantic fluency (the ability to access words within a specific conceptual category) can impair the verbal mediation of emotions (Shao et al., 2014), leading to frustration during group therapy and increasing the likelihood of program abandonment. Although these last studies were not conducted with IPV perpetrators, they reinforce the need to incorporate a comprehensive assessment covering all of these neuropsychological domains to determine whether this risk profile in other populations also applies to IPV perpetrators in explaining treatment dropout.

With all of this in mind, the present study aims to examine the predictive power of various neuropsychological variables, including IQ, cognitive flexibility, planning ability, verbal fluency, attention, and working memory, on the premature dropout of IPV perpetrators enrolled in an intervention program. This analysis was conducted while controlling for sociodemographic variables previously identified in the scientific literature as relevant to dropout among IPV perpetrators (e.g., age, educational level, nationality, working status) (Cunha et al., 2024; Jewell & Wormith, 2010; D. B. Wilson et al., 2021) and alcohol misuse (Lila et al., 2020; Nguyen et al., 2023). In line with previous research in the field of neuropsychological variables (Cunha et al., 2024; Nguyen et al., 2023; Romero-Martínez et al., 2023, 2024), we expected all included neuropsychological variables (IQ, cognitive flexibility, planning abilities, verbal fluency, attention, and working memory) to show an association with dropout among IPV perpetrators, even after controlling for potential confounding variables. Dropout was expected to be particularly linked to executive functioning (e.g., cognitive flexibility, planning abilities, and verbal fluency) and IQ alterations. By elucidating the cognitive or neuropsychological underpinnings of treatment resistance or abandonment, we seek to provide a foundation for more effective intervention strategies that diminish the risk of attrition in IPV perpetrators, which in turn, could mitigate the risk of recidivism and enhance public safety.

## 2. Materials and Methods

### 2.1. Participants

The main objective of this study was to conduct a regression model to examine dropout based on several neuropsychological variables including specific sociodemographic variables as covariates. To calculate the necessary sample size for the generalized regression models, we considered an anticipated effect size (f2) of 0.15, a desired statistical power level of 0.95, approximately a maximum of 15 predictors (main predictors and covariates), and a probability level of 0.05. The calculation of power analysis determined a minimum sample size of 200 participants for our study.

The recruitment process began by explaining the study to men whose IPV sentences had been suspended within the last two years, contingent on attending a university-based intervention program (Lila et al., 2020). Of the 491 men who provided written informed consent, 182 were excluded due to incomplete measurements or missing data regarding dropout or several of the main variables for this study. Specifically, missing values primarily occurred in some neuropsychological tasks due to participants failing to complete the entire testing session. Additionally, sociodemographic data or court records regarding exact dropout classification were missing. To ensure that this sample reduction did not introduce systematic attrition bias, a comprehensive series of sensitivity analyses was conducted. Accordingly, group comparisons did not reveal statistical significant differences between the included and excluded participants regarding age (*t* = −0.431, *p* = 0.666), marital status (*X*^2^ = 3.58, *p* = 0.311), nationality (*X*^2^ = 0.11, *p* = 0.994), educational level (*X*^2^ = 6.40, *p* = 0.094), employment status (*X*^2^ = 0.097, *p* = 0.735), or alcohol misuse (*t* = 0.677, *p* = 0.250).

The final sample consisted of 309 participants who completed all research measures (see Table 1).

To be eligible for the study, participants had to be at least 18 years of age, free of mental disorders (e.g., schizophrenia), and have no previous history of severe traumatic brain injury (e.g., loss of conscience from minutes to hours, need for posterior rehabilitation or suffering from severe sequelae) or stroke-related brain damage. The sample consisted of IPV perpetrators who had received suspended sentences of less than two years, contingent upon their participation in a court-mandated intervention program specifically designed for them. This intervention followed a psychoeducational and community-based approach (Lila et al., 2020).

The research was conducted in accordance with the Declaration of Helsinki and received formal approval from the University Ethics Committee (Protocol Codes: H1515749368278 and 2024-PSILOG-3527732).

### 2.2. Instruments

A set of neuropsychological tests, comprising relevant cognitive domains for IPV perpetrators (see Table 2), was used in this study (Romero-Martínez et al., 2024).

General verbal and non-verbal intelligence were assessed using the Kaufman Brief Intelligence Test (K-BIT), consisting of the Vocabulary and Matrices subtests (Kaufman & Kaufman, 1997).

Working memory was measured using the total score (forward and backward digits) of the Wechsler Intelligence Scale-III (WAIS-III) Digits subscale (Wechsler, 1999).

The Conners’ Continuous Performance Test-III (CPT-III) was used to assess attention, impulsivity, and processing speed; performance was quantified through total reaction time, omissions, commissions, and perseverations (Conners, 2014).

Verbal fluency was evaluated using both phonemic (F-A-S) and semantic (animal naming) tasks, where higher scores reflect superior fluency (Del Ser Quijano et al., 2004).

Cognitive flexibility was measured with the Wisconsin card sorting test (WCST), specifically analyzing the number of trials, non-perseverative and perseverative errors, completed categories, and failures to maintain set (Heaton et al., 1993).

Finally, planning abilities were assessed using the Key Search Test from the Behavioral Assessment of the Dysexecutive Syndrome (BADS), recording the total score and time spent on planning and execution (B. A. Wilson et al., 1996).

Alcohol misuse was assessed using the Spanish version (Contell-Guillamón et al., 1999) of the Alcohol Use Disorders Identification Test (AUDIT; Saunders et al., 1993). This self-report instrument comprises 10 items scored on a Likert scale ranging from 0 (never) to 4 (daily or almost daily), yielding a total score between 0 and 40. In the present study, the scale demonstrated acceptable internal consistency, with a Cronbach’s alpha of 0.84.

Treatment compliance was assessed with a dichotomous variable to categorize participants as either completers (0), those who finished the entire program, or dropouts (1), those who discontinued the intervention prematurely. That is, those who did not complete the 35-week intervention.

### 2.3. Procedure

Prior to enrollment, participants were formally briefed on the study’s objectives and assured that participation would remain strictly confidential and have no impact on their legal status. Data collection was conducted across two laboratory sessions at the Faculty of Psychology. Before conducting the first session, an initial screening interview was conducted to exclude individuals with physical or mental health conditions that might interfere with the intervention process. It was carried out by two trained psychologists with extensive experience in the field of IPV perpetrator interventions. For men who expressed interest in the study and met the eligibility criteria, researchers conducted a semi-structured interview during the first session to record sociodemographic data and information regarding alcohol misuse. The second session took place the following day between 10:00 a.m. and 2:00 p.m. to mitigate the potential confounding effects of circadian rhythms and fatigue that could influence neuropsychological assessment. This 90-min (approximately) session included a comprehensive set of neuropsychological tests (see Table 2) and was conducted before starting the intervention program.

The intervention consisted of a manualized, evidence-based program administered by experienced psychotherapists (see Lila et al., 2020, for a detailed description). Treatment compliance, specifically dropout rates, was recorded throughout the 35-week program (one 2-h session per week).

### 2.4. Data Analysis

Before conducting a logistic regression model, with dropout as the dependent variable, neuropsychological variables as independent variables, and sociodemographic and alcohol misuse as covariates, partial correlation analyses were performed to explore the relationships between neuropsychological variables (predictors or independent variables) and the outcome variable (dropout). Sociodemographic characteristics and alcohol misuse were included as covariates to control for potential confounding effects. Employing partial correlations allows for identifying which subscales of each neuropsychological test are highly correlated with dropout to obtain the best model that explains dropout among IPV perpetrators. Furthermore, it avoids multicollinearity in the regression model. Covariates were included in step 0, adding the main predictors in step 1. Additionally, we provided the variance inflation factor (VIF) to assess multicollinearity. To analyze classification performance, we employed two metrics such as the model accuracy and the area under the receiver operating characteristic (ROC) curve (AUC). These metrics offer additional information regarding the predictive accuracy and the discriminative power of the model.

All analyses were performed using IBM SPSS Statistics (Version 29.0), with the significance level set at *p* = 0.05.

## 3. Results

Regarding treatment compliance, of the 309 IPV perpetrators who met the inclusion criteria and completed the baseline assessment, 81 participants (26%) dropped out before the end of the 35-week intervention. Therefore, 228 participants (74%) successfully completed the full manualized program, providing a robust distribution of the data for the subsequent regression analyses used to identify neuropsychological predictors of dropout.

Results of partial correlations are summarized in Table 3. Specifically, dropout was negatively associated with IQ, nonverbal IQ, WCST categories completed, FAS phonemic test, and Key Search Total Executing Time. Conversely, dropout was positively associated with WCST perseverative errors and CPT-3 omissions. In order to reduce the number of variables in the regression model and avoid multicollinearity, we only included the variables from each test that were highly correlated with dropout (nonverbal IQ, WCST perseverative errors, FAS phonemic test, Key Search Total Executing Time, and CPT-3 omissions).

Regarding the main objective of this study, we hypothesized that greater neuropsychological alterations would be associated with higher dropout rates while covariating potential confounding variables. The omnibus test showed that the model including the neuropsychological variables was significant (Chi square = 66.49, *p* < 0.001), indicating that some variables included in the model had a significant impact on the dependent variable. The Cox & Snell R^2^ was 0.194, and Nagelkerke’s R^2^ was 0.283. The Cox & Snell R^2^ indicates that the model explained a small proportion of dropout, whereas Nagelkerke’s R^2^ suggests a moderate relationship with the dependent variable (dropout). Among the neuropsychological variables included, only four appeared to be relevant for explaining dropout among IPV perpetrators. These variables were nonverbal IQ (Odds ratio = 0.973; 95% CI 0.952 to 0.995), WCST perseverative errors (Odds ratio = 1.031; 95% CI 1.013 to 1.049), Key Search Total Executing Time (Odds ratio = 0.980; 95% CI 0.967 to 0.994), and CPT-3 omissions (Odds ratio = 1.039; 95% CI 1.000 to 1.080). In particular, WCST perseverative errors and CPT-3 omissions appeared to be relevant for explaining dropout, even when controlling the effect of potential confounding variables (Table 4).

We employed the VIF to analyze multicollinearity. All analyzed variables exhibited VIF values ranging from 1.033 to 1.522. This confirmed the absence of multicollinearity and ensured the stability of the model.

Regarding the model’s predictive performance, it demonstrated a robust overall discrimination capability, achieving a global AUC of 0.785 and an overall model quality of 0.73. Specifically, the model allowed distinguishing between IPV perpetrators who dropped out of the program and those who completed it.

## 4. Discussion

The present study aimed to investigate whether neuropsychological impairments, specifically in terms of IQ, cognitive flexibility, planning abilities, verbal fluency, working memory, and attention, serve as significant predictors of treatment dropout among IPV perpetrators. Our findings suggest an association between treatment dropout and both nonverbal reasoning and certain executive alterations, such as low cognitive flexibility, planning abilities, and inattention. Specifically, these variables emerged as relevant markers for intervention discontinuation, even after controlling for sociodemographic risk factors and alcohol misuse. Our findings seem particularly relevant for the group of IPV perpetrators who dropped out, as revealed by the accuracy and the AUC of the models. However, the predictive value of these variables should be interpreted with caution; although they were significantly associated with dropout, the variables alone did not fully explain the phenomenon. They could, therefore, be considered complementary to other variables previously identified in this field of research, such as sociodemographic or psychological variables.

The most salient finding in our regression model was the role of WCST in intervention dropout rates among IPV perpetrators (OR = 1.031, 95% IC 1.013 to 1.049), which is congruent with previous findings in this field of research (Romero-Martínez et al., 2023). Higher rates of perseverative errors significantly increased the odds of dropping out. This suggests that perpetrators who exhibit mental rigidity, understood as the inability to shift cognitive sets or adapt to new rules, struggle the most with the demands of a therapeutic intervention. For example, these individuals may find it difficult to internalize alternative conflict-resolution strategies or abandon deeply ingrained hostile schemas. This mental perseveration likely leads to frustration during the psychoeducational process, ultimately resulting in premature abandonment of the program. Moreover, shorter execution times in the Key Search Test (related to worse performance in this test) were associated with a higher probability of dropout. This may reflect a profile of impulsivity or a lack of systematic planning. Individuals who rush through tasks without adequate cognitive deliberation may likely exhibit the same impulsive behavioral patterns in their personal lives, increasing the likelihood of a sudden, non-reflective decision to leave the court-mandated program. This is further supported by the positive correlation between dropout and omission errors during sustained attention tasks, which have been typically associated with lapses in sustained attention and inhibitory control. Therefore, rather than generalized cognitive impairment, our results signaled the importance of a selective executive profile linked to premature abandonment of the intervention. The prominent role of perseverative errors and diminished planning abilities may reflect cognitive factors associated with how these individuals process program demands, such as a possible difficulty in shifting cognitive sets and adapting to new rules.

Current results also highlighted that lower nonverbal IQ was a significant predictor of dropout. This signals the importance of abstract problem-solving in treatment adherence. IPV perpetrators with lower nonverbal reasoning abilities may face difficulties processing the complex social scenarios and cognitive-behavioral tools introduced in interventions, making the sessions feel overwhelming or irrelevant to their lived experience. Hence, our results aligned with studies reporting significant correlations between certain cognitive domains and treatment adherence (psychotherapy or medication), as shown in patients with schizophrenia (Wojtalik et al., 2023; Zhu et al., 2023), HIV patients (Becker et al., 2011), individuals with bipolar disorder (Fagan et al., 2015), and those with drug use disorder (Stimmel et al., 2018). Our results reinforce the potential value of assessing neuropsychological variables prior to intervention, offering avenues for future research to test whether tailored treatments or adjunct modules can improve attendance and adherence.

Despite the use of a comprehensive battery of neuropsychological tests (Table 2) based on the conclusions published in a meta-analysis of neuropsychological alterations in IPV perpetrators (Romero-Martínez et al., 2024) and the scientific literature focused on neuropsychological predictors of dropout in different populations (Becker et al., 2011; Fagan et al., 2015; Romero-Martínez et al., 2023; Stimmel et al., 2018; Wojtalik et al., 2023; Zhu et al., 2023), only a subset of these measures demonstrated a significant association with treatment dropout among IPV perpetrators. This selective relationship suggests that intervention discontinuation is specifically linked to certain neuropsychological or cognitive domains rather than a generalized cognitive impairment. Variables that did not reach statistical significance, such as verbal IQ, working memory, or semantic fluency, may play a secondary role or be less critical for the specific demands of the therapeutic process. This selectivity suggests that the endurance required to complete an IPV program shares a stronger association with dynamic fluid reasoning and real-time behavioral regulation than with linguistic abilities or short-term informational storage. IPV perpetrators with lower nonverbal reasoning may find the abstract, multi-step socio-cognitive tools introduced in therapy overwhelming. Therefore, the lack of significance in more passive cognitive measures implies that interventions do not fail because participants cannot comprehend or remember the words spoken in therapy, but because they lack the executive flexibility to operationalize those concepts when their cognitive schemas are challenged. Furthermore, the lack of association in some scales could be attributed to the high degree of overlap between certain cognitive functions, where more sensitive indicators (e.g., WCST perseverative errors) captured the variance that more general measures failed to detect. Furthermore, the absence of a significant association in some tests may be explained by certain methodological issues related to the psychometric properties of these instruments or the use of tests that yield only a single total score. Additional research could consider using alternative neuropsychological instruments that capture memory and language domains.

The inclusion of demographic variables and alcohol misuse as covariates was consistent with previous literature, which identified alcohol misuse and employment status as strong predictors of dropout (Cunha et al., 2024; Jewell & Wormith, 2010; D. B. Wilson et al., 2021). However, the fact that neuropsychological variables remained significant after controlling for these variables suggests that cognitive deficits provide unique explanatory power beyond substance misuse or socioeconomic instability. Nevertheless, alcohol misuse and employment status should not be interpreted as independent factors that explain dropout. Rather, the interaction between neuropsychological performance and situational factors warrants closer examination. Alcohol misuse may act as a catalyst that exacerbates underlying cognitive impairments, further compromising behavioral regulation and decision-making processes (Romero-Martínez et al., 2024). Moreover, in cases where IPV perpetrators are employed, professional responsibilities may create logistical conflicts with therapy attendance. In this context, cognitive alterations, specifically deficits in flexibility and planning, may explain why these men struggle to navigate such conflicting demands. Those with better executive control are more likely to be capable of organizing their schedules and managing the frustration of balancing work and treatment. Conversely, perpetrators with significant cognitive rigidities may perceive these external pressures as insurmountable obstacles, ultimately choosing to prioritize immediate job demands over long-term intervention goals, which significantly increases the risk of premature dropout. Hence, our study reinforces moving beyond traditional paradigms to explain IPV dropout, highlighting that neuropsychological variables are significantly associated with treatment discontinuation. In particular, executive alterations (e.g., mental rigidity and compromised planning abilities), together with inattention and limitations in nonverbal reasoning, retained their predictive validity even after controlling for robust covariates such as alcohol misuse and socioeconomic instability.

Findings from this study present considerable implications for IPV intervention designs and suggest moving beyond a profile of IPV perpetrators mainly based on sociodemographic, violence typology, criminal history, and psychological variables (Giesbrecht et al., 2026), without considering other variables such as neuropsychological factors. Our study found that neuropsychological variables were significantly associated with IPV perpetrators’ treatment adherence, suggesting that therapeutic failure in IPV programs is not merely a consequence of external resistance or lack of motivation, but is also related to IPV perpetrators’ neuropsychological profile. However, it is important to note that the capacity of neuropsychological variables to explain dropout was relatively low. Therefore, these variables should not be viewed as exclusive predictors, but rather as a complementary framework that enriches existing sociodemographic and psychological perspectives on treatment discontinuation in this population. Standardized one-size-fits-all programs, at least for a small group of IPV perpetrators with these cognitive alterations, may be ineffective. To integrate these findings into practice without overburdening clinical resources, we suggest judicial protocols to benefit from a rapid screening stage rather than administering a lengthy set of neuropsychological tests to every IPV perpetrator. Although we provided a comprehensive neuropsychological set of tests, the shorter versions of the Wisconsin card sorting test could be used alongside computerized attention tests to build a brief assessment. In cases in which the IPV perpetrator shows slight deficits or conversely, if the tests do not offer relevant information, an exhaustive assessment could be carried out. Thus, as a tentative hypothesis for future research, early screening during the intake process could be explored as a tool to predict IPV perpetrators with a higher risk of dropout, using various screening tools depending on contextual and individual demands.

Regarding intervention delivery within rigid court-mandated structures, future studies could evaluate the utility of introducing brief adjunct modules. For example, for individuals with high perseverative errors, a pre-treatment phase of 2-to-3 sessions focused on improving cognitive flexibility and motivational interviewing before entering the main group could be tested to see if it enhances later adherence to the psychoeducational program. Moreover, a simplification or adaptation of abstract concepts with low-cost instructional modifications could help those IPV perpetrators with lower nonverbal IQ to ensure that the intervention remains accessible and engaging to those men. This could be conducted by employing concrete visual aids and breaking down multi-step conflict-resolution tools into shorter, sequential behavioral steps. In fact, it would be interesting to explore whether the inclusion of adjunct cognitive training targeting impaired cognitive domains within batterer intervention programs would enhance treatment attendance. In this sense, a previous study concluded that adding cognitive training to a standard intervention program led to significant improvements in processing speed and cognitive flexibility among IPV perpetrators, which in turn, entailed a lower risk of recidivism after treatment (Romero-Martínez et al., 2022). Given that these improvements were moderate, these variables or the implementation of adjunct cognitive training programs cannot be considered as a substitute for current interventions, they should be considered complementary. Using them alongside current interventions may help reduce dropout and recidivism rates.

Despite the significance of the findings based on a considerable sample of IPV perpetrators and a comprehensive set of neuropsychological tests, several limitations should be acknowledged. The first limitation relates to the absence of specific tests to control for malingering or the fabrication of responses, such as the Test of Memory Malingering (TOMM). It is possible that some IPV perpetrators may have exaggerated the presence of cognitive deficits, which could mask the true extent of their cognitive impairments. Second, the use of self-report measures to assess alcohol misuse might be subject to social desirability bias or retrospective recall inaccuracies, which are common in forensic populations. Third, while the sample size was relatively large, the participants were exclusively IPV perpetrators with suspended sentences of less than two years and no prior criminal records; therefore, the generalizability of these results to higher-risk offenders or those in prison settings may be limited. Fourth, although we controlled for a wide range of confounding variables, including alcohol misuse and sociodemographic factors, the correlational and cross-sectional nature of the study precludes the establishment of definitive causal relationships between cognitive deficits and dropout. Thus, future research should incorporate longitudinal follow-ups to better understand how executive dysfunctions explain treatment dropout. Future research could also explore whether other cognitive domains (e.g., declarative memory, learning, inhibitory control, among others) are relevant for explaining dropout. Additionally, to complement this dichotomous assessment of dropout, it would be important to introduce other quantitative measurements such as the number of treatment sessions. Furthermore, selecting neuropsychological variables based on scientific literature and significant partial correlations risks inflating the explanatory value of the final predictive regression model. In fact, the selection of the neuropsychological variables was largely data-driven, as we selected the variables included in the regression analysis based on their prior partial correlations with the outcome (dropout). This statistical approach carries a substantial risk of overfitting, which may have inflated the apparent relevance and predictive power of our final model. Finally, the clinical implications based on our results completely lack external validation in independent cohorts. Therefore, without testing these predictive patterns in other samples, the generalizability and usefulness of our findings remain unverified.

## 5. Conclusions

In summary, within the limits of our observational design, this study suggests that dropping out of an IPV intervention may be linked, among other factors, to the IPV perpetrator’s neuropsychological profile. Specifically, our findings indicate that certain cognitive domains, such as nonverbal IQ, cognitive flexibility, planning abilities, and attention might be relevant for predicting treatment dropout among IPV perpetrators, at least within a considerable subgroup. In this sense, our study reinforces the need for the screening of neuropsychological variables including executive functions and fluid intelligence before starting interventions. The clinical implications of our study remain speculative, as external validation in independent cohorts is necessary; however, these variables should be considered complementary to current assessments and interventions rather than substitutes, offering additional support to traditional programs. This would allow us to better characterize the neuropsychological profile of IPV perpetrators and to explore whether implementing cognitive training or adapting psychoeducational modules for high-risk individuals could potentially enhance treatment adherence. Whether treatment retention can be improved by moving beyond sociodemographic profiles and addressing the underlying cognitive rigidities associated with disrupted rehabilitation remains a question that requires empirical verification through randomized controlled trials. In any case, we need to be cautious regarding the capacity of these variables to explain a complex phenomenon such as dropout. Ultimately, these recommendations require external validation in independent cohorts before they can be safely implemented in direct clinical or judicial practice.

## Figures and Tables

**Table 1:** Descriptive characteristics of intimate partner violence perpetrators (mean, standard deviation and percentages).

Variables		IPV Perpetrators (*n* = 309)
Age (years)		41.66 ± 11.56
Marital status (%)	Married	24%
	Single/separated/divorced/widowed	76%
Nationality (%)	Spanish	76%
	Latin-American	9%
	Others	15%
Educational level (%)	Primary	43.5%
	Secondary	45.8%
	Upper level	10.7%
Employment status (%)	Yes	65.8%
	No	34.2%
Alcohol misuse		5.57 ± 6.59

**Table 2:** Neuropsychological tests listed in order of administration.

Neuropsychological Test and Subscale(s) Employed	Cognitive Domain	Time of Administration (Instructions + Administration)
Kaufman Brief Intelligence Test (K-BIT)	IQ (General verbal and non-verbal intelligence)	30 min
Digit Subscale of the Wechsler Intelligence Scale-III	Working memory	3 min
Conners’ Continuous Performance Test-III (CPT-III)	Sustained attention and vigilance	15 min
Phonemic (F-A-S) and Semantic (animal naming) task	Verbal fluency	5 min
Wisconsin Card Sorting Test (WCST)	Cognitive flexibility	30 min
Key Search Test	Planning abilities	7 min

**Table 3:** Partial correlations between neuropsychological variables and dropout including sociodemographic and alcohol misuse as covariates.

Neuropsychological Variables	Dropout	1	2	3	4	5	6	7	8	9	10	11	12
1. IQ	−0.201 ***												
2. Nonverbal IQ	−0.230 ***	0.815 ***											
3. Verbal IQ	−0.111	0.479 ***	0.310 ***										
4. WCST perseverative errors	0.242 ***	−0.281 ***	−0.224 ***	−0.187 ***									
5. WCST categories completed	−0.125 *	−0.021	0.172 **	0.099	−0.328 ***								
6. FAS phonemic test	−0.161 **	0.521 ***	0.430 ***	0.381 ***	−0.210 ***	0.149 *							
7. FAS semantic test	−0.020	0.309 ***	0.278 ***	0.153 *	−0.108	0.033	0.472 ***						
8. Key Search test total score	−0.037	0.294 ***	0.224 ***	0.176 **	−0.096	−0.046	0.242 ***	0.121 *					
9. Key Search Total Planning Time	−0.096	0.038	0.016	−0.007	−0.039	0.020	−0.020	0.026	−0.058				
10. Key Search Total Executing Time	−0.116 *	0.101	0.078	0.018	0.060	−0.016	−0.019	0.008	0.119 *	0.352 ***			
11. CPT-3 omissions	0.160 **	−0.265 ***	−0.202 ***	−0.158 **	0.166 **	−0.060	−0.185 **	−0.077	−0.046	0.018	0.062		
12. CPT-3 perseverations	0.102	−0.186 ***	−0.171 **	−0.107	0.042	−0.05	−0.0132 *	0.023	−0.009	0.102	0.053	0.270 ***	
13. Digit test total score	−0.083	0.320 ***	0.288 ***	0.177 **	−0.188 ***	0.153 **	0.383 ***	0.238 ***	0.048	0.052	−0.018	−0.153 **	−0.145 *

Note. CPT-3: Conners Continuous Performance Test-3; IQ: intelligence quotient; WCST: Wisconsin card sorting test. * *p* < 0.05; ** *p* < 0.01; *** *p* < 0.001.

**Table 4:** Regression model explaining dropout among IPV perpetrators based on neuropsychological variables, covariating sociodemographic variables, and alcohol misuse.

Effect	B	SE B	*Exp* (*β*)	95% CI for *Exp* (*β*)	
				LL	UL
Step 0					
Constant	−1.343	0.831	0.261		
Age	−0.001	0.013	0.999	0.973	1.025
Marital status	−0.114	0.133	0.892	0.687	1.157
Nationality	0.051	0.185	1.053	0.732	1.514
Educational level	−0.372	0.190	0.689	0.475	0.999
Employment status	1.035	0.286	2.822 ***	1.613	4.939
Alcohol misuse	0.261	0.020	1.035	0.995	1.077
Step 1					
Constant	0.794	1.242	2.212		
Age	−0.017	0.014	0.983	0.955	1.011
Marital status	−0.137	0.146	0.872	0.655	1.162
Nationality	−0.012	0.205	0.988	0.661	1.476
Educational level	−0.068	0.223	0.934	0.603	1.447
Employment status	1.102	0.317	3.010 ***	1.618	5.601
Alcohol misuse	0.037	0.023	1.038	0.992	1.086
Nonverbal IQ	−0.027	0.011	0.973 *	0.952	0.995
WCST perseverative errors	0.030	0.009	1.031 ***	1.013	1.049
FAS phonemic test	−0.009	0.016	0.991	0.960	1.023
Key Search Total Executing Time	−0.020	0.007	0.980 **	0.967	0.994
CPT-3 omissions	0.039	0.020	1.039 *	1.000	1.080

Note. CPT-3: Conners Continuous Performance Test-3; IPV: intimate partner violence; IQ: intelligence quotient; WCST: Wisconsin card sorting test. * *p* < 0.05; ** *p* < 0.01; *** *p* < 0.001.

## Data Availability

Due to the characteristics of the sample and for data protection reasons, all data supporting the findings of this study are available from the corresponding author upon request. Access to the data is granted solely for academic and research purposes, and all requests will be reviewed to ensure compliance with privacy and ethical considerations.

## Abbreviations

The following abbreviations are used in this manuscript:

BADS	Behavioral Assessment of the Dysexecutive Syndrome
CI	Confidence Interval
CPT-III	Conners’ Continuous Performance Test-III
IPV	Intimate Partner Violence
IQ	Intelligence Quotient
KBIT	Kaufman Brief Intelligence Test
LL	Lower Limit
UL	Upper Limit
WAIS-III	Wechsler Intelligence Scale-III
WCST	Wisconsin Card Sorting Test
